# Real-time optical diagnosis of gastric cancer with serosal invasion using multiphoton imaging

**DOI:** 10.1038/srep31004

**Published:** 2016-08-08

**Authors:** Jun Yan, Yu Zheng, Xiaoling Zheng, Zhangyuanzhu Liu, Wenju Liu, Dexin Chen, Xiaoyu Dong, Kai Li, Xiumin Liu, Gang Chen, Jianping Lu, Jianxin Chen, Shuangmu Zhuo, Guoxin Li

**Affiliations:** 1 Department of General Surgery, Nanfang Hospital, Southern Medical University, Guangzhou, Guangdong, 510515, P. R. China; 2 Fujian Provincial Key Laboratory for Photonics Technology & Key Laboratory of Optoelectronic Science and Technology for Medicine of Ministry of Education, Fujian Normal University, Fuzhou, 350007, P. R. China; 3 Department of Surgery, Fujian Provincial Hospital, Teaching Hospital of Fujian Medical University, Fuzhou, Fujian, 350001, P. R. China; 4 Department of Pathology, Fujian Provincial Tumor Hospital, Teaching Hospital of Fujian Medical University, Fuzhou, Fujian, 350014, P. R. China; 5 Department of Surgery, Fujian Provincial Tumor Hospital, TeachingHospital of Fujian Medical University, Fuzhou, Fujian, 350014, P. R. China

## Abstract

A real-time optical biopsy, which could determine tissue histopathology, would be of extraordinary benefit to staging laparoscopy for gastric cancer with serosal invasion (T4) that requires downstage treatment. We investigated the feasibility of using multiphoton imaging to perform a real-time optical diagnosis of gastric cancer with or without serosal invasion. First, a pilot study was performed to establish the optical diagnostic features of gastric cancer with or without serosal invasion using multiphoton imaging compared with hematoxylin-eosin staining and Masson’s trichrome staining. Second, a blinded study was performed to compare the diagnostic sensitivity, specificity, and accuracy of multiphoton imaging and endoscopic ultrasonography (EUS) for T4 gastric cancer. In the pilot study, multiphoton imaging revealed collagen loss and degradation and cellular and nuclear pleomorphism in gastric cancer with serosal invasion. The collagen content in gastric cancer with or without serosal invasion was 0.36 ± 0.18 and 0.79 ± 0.16 (*p* < 0.001), respectively. In the blinded study, the sensitivity, specificity, and accuracy of EUS and multiphoton imaging for T4 gastric cancer were 70% and 90% (*p* = 0.029), 66.67% and 96.67% (*p* = 0.003), and 68.33% and 93.33% (*p* = 0.001), respectively. It is feasible to use multiphoton imaging to make a real-time optical diagnosis of gastric cancer with or without serosal invasion.

Staging laparoscopy has a significant impact on decisions regarding the treatment plan for patients with advanced gastric cancer^1^,^2^. For gastric cancer with serosal invasion (T4), neoadjuvant therapy is required to downstage the tumor^3^,^4^. Currently, there is no instrument available to perform real-time *in-situ* histopathological diagnoses for gastric cancer with serosal invasion. Endoscopic ultrasound (EUS) and computed tomography (CT) are inadequate for identifying individual T stages^5^. Therefore, the availability of a non-invasive optical diagnosis that could provide a real-time *in-situ* analysis comparable to gold standard hematoxylin-eosin (H&E) staining histopathology would be of extraordinary benefit to staging laparoscopy and subsequent neoadjuvant therapy of gastric cancer. Multiphoton imaging, based on advancements in the field of non-linear optics and femtosecond lasers, could provide real-time detailed information regarding tissue architecture and cell morphology in live tissue using a combination of two-photon autofluorescence (TPA) from cells or elastic fibers and second harmonic generation (SHG) signaling from collagen^6^,^7^,^8^. Natural intrinsic fluorophores that are abundantly present in most cells include reduced nicotinamide adenine dinucleotide (NADH) and flavin adenine dinucleotides (FAD); these fluorophores are also present in structural proteins such as elastic fibers^9^. The purpose of this study was to evaluate the feasibility of using multiphoton imaging to make a real-time optical diagnosis of gastric cancer with or without serosal invasion.

## Results

### Patient demographics and cancer characteristics

Twenty patients with gastric cancer in the pilot study and sixty patients with gastric cancer in the blinded study underwent gastrectomy. The patient demographics and cancer characteristics are summarized in Tables 1 and 2.

### In the pilot study, multiphoton imaging showed significant differences between gastric cancer without serosal invasion and gastric cancer with serosal invasion

**Figure 1:**
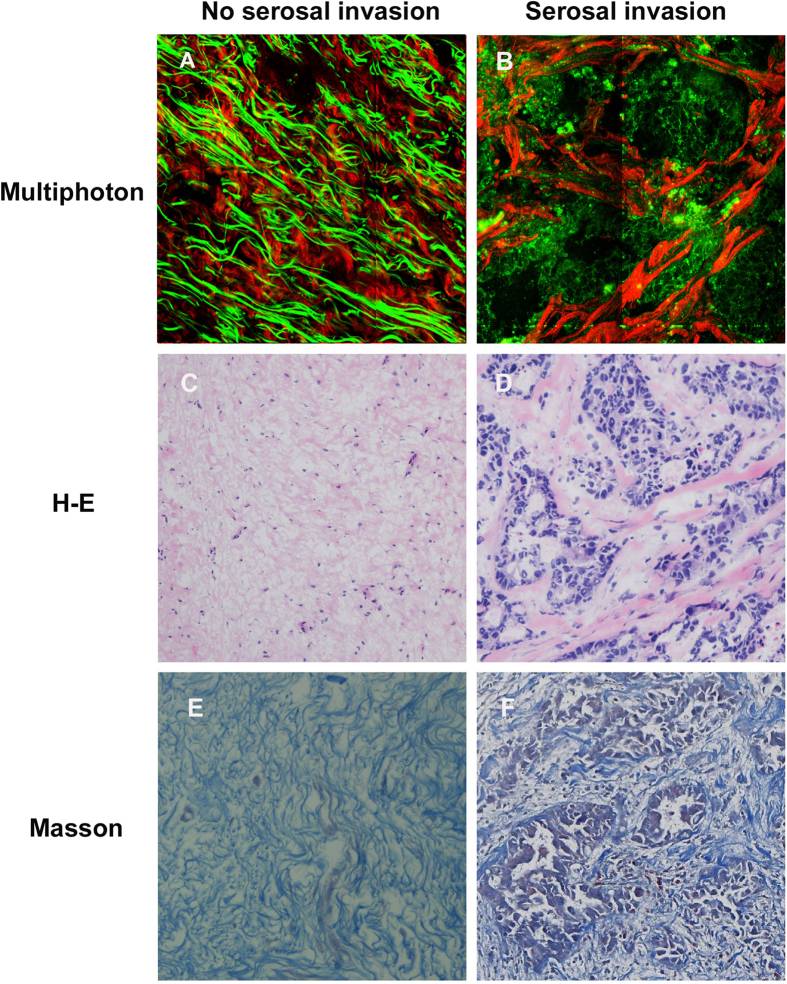
Comparisons between the multiphoton images, H&E staining images, and Masson’s trichrome staining images in gastric cancer with or without serosal invasion using multiphoton imaging. (**A**) The multiphoton image (63×) of fresh, unfixed, and unstained gastric cancer without serosal invasion revealed a regular collagen structure (red). (**B**) The multiphoton image (63×) of fresh, unfixed, and unstained gastric cancer with serosal invasion demonstrates an irregular collagen structure (red) and cellular and nuclear pleomorphism (green). Cancer cells were characterized by an irregular size and shape, enlarged nuclei, and an increased nuclear-cytoplasmic ratio. The SHG signal shows that the collagen content is significantly decreased and that degradation of the collagen structure is increased in the cancerous area. (**C**) The corresponding H&E image (20×) of fixed, stained gastric cancer without serosal invasion shows a regular collagen structure in the normal serosa. (**D**) The corresponding H&E image (20×) of fixed, stained gastric cancer with serosal invasion shows irregular collagen structure and cellular and nuclear pleomorphism, which corresponds to the multiphoton image. (**E**) The corresponding Masson’s trichrome staining image (20×) shows a regular collagen structure in gastric cancer without serosal invasion. (**F**) The corresponding Masson’s trichrome staining image (20×) shows an irregular collagen structure and a significant collagen loss in gastric cancer with serosal invasion.

**Figure 2:**
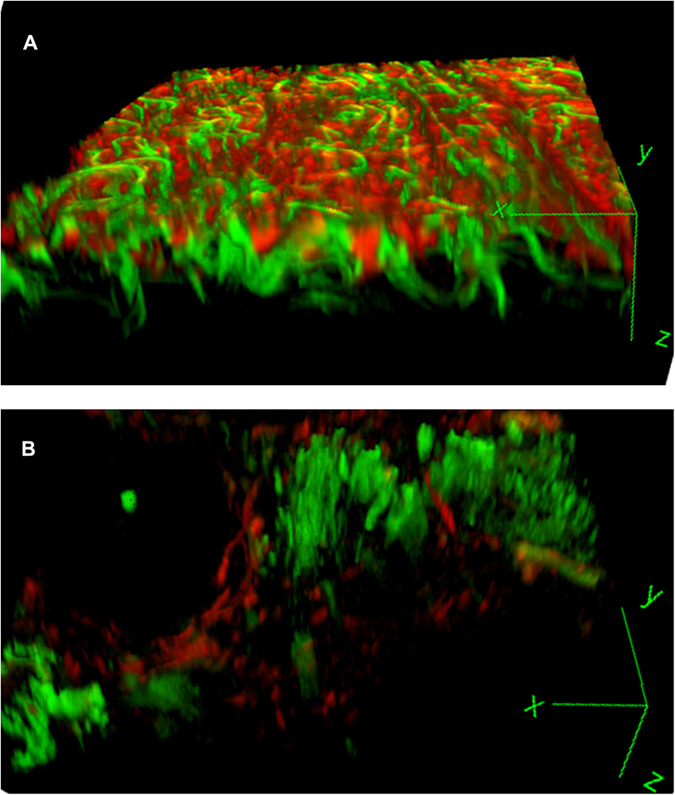
Multiphoton 3D-stacking imaging. (**A**) The multiphoton 3D-stacking imaging (63×) clearly shows a regular serosal collagen arrangement (red) in gastric cancer without serosal invasion. (**B**) The multiphoton 3D-stacking imaging (63×) shows an irregular collagen structure (red) and serosal collagen loss in gastric cancer with serosal invasion. The SHG signal shows that the collagen content is significantly decreased and that the degradation of the collagen structure is increased in the cancerous area.

Each gastric specimen was examined using multiphoton imaging. The multiphoton images were acquired using the following two channels: broadband autofluorescence from cells and SHG from tissue collagen. The peak multiphoton autofluorescence intensity was detected in serosa excited at 800 nm. There were significant differences between gastric cancer without serosal invasion and gastric cancer with serosal invasion. In gastric cancer without serosal invasion, the multiphoton imaging revealed a regular collagen structure (Fig. 1A). These features corresponded to the H&E stained images (Fig. 1C) and the Masson’s trichrome stained images (Fig. 1E). In gastric cancer with serosal invasion, the multiphoton imaging demonstrated an irregular collagen structure, collagen loss, and cellular and nuclear pleomorphisms (Fig. 1B). The cancer cells were characterized by an irregular size and shape, enlarged nuclei, and an increased nuclear-cytoplasmic ratio. These same details of tissue architecture and cell morphology were similar to the H&E stained images (Fig. 1D) and the Masson’s trichrome stained images (Fig. 1F). Moreover, the multiphoton 3D-stacking imaging clearly showed a regular collagen arrangement in gastric cancer without serosal invasion (Fig. 2A) and collagen loss in gastric cancer with serosal invasion (Fig. 2B). SHG signals could provide quantitative features to effectively evaluate the changes in collagen in gastric cancer with or without serosal invasion. The collagen content and the collagen structure were extracted. In this work, the collagen content in gastric cancer without serosal invasion was 0.79 ± 0.16, and the collagen content in gastric cancer with serosal invasion was 0.36 ± 0.18, demonstrating collagen loss in gastric cancer with serosal invasion. The correlation value of collagen structure in gastric cancer without serosal invasion was 0.21 ± 0.05 and the value in gastric cancer with serosal invasion is 0.69 ± 0.12, supporting the idea that collagen structure degradation increases in gastric cancer with serosal invasion. Multiphoton imaging showed significant differences between gastric cancer without serosal invasion and gastric cancer with serosal invasion, and multiphoton images were comparable to H&E stained images and Masson’s trichrome stained images. The optical diagnostic features of gastric cancer with or without serosal invasion using multiphoton imaging are shown in Table 3.

### In the blinded study, multiphoton imaging significantly improved the diagnostic accuracy for T4 gastric cancer compared with EUS

Using the multiphoton diagnostic features of gastric cancer with or without serosal invasion established in the pilot study, we performed a blinded study to test the sensitivity, specificity, and accuracy of multiphoton imaging by investigating 60 patients with gastric cancer. Each patient underwent preoperative endoscopic ultrasonography (EUS) and then received radical gastrectomy with D2 lymphadenectomy. The serosal side of each sample was examined using multiphoton imaging, and then each sample went through routine pathological procedures. The multiphoton images were analyzed and diagnosed by the attending pathologist who established the multiphoton diagnostic features. The EUS doctor and the attending pathologist who analyzed the multiphoton images were blinded to the final H&E pathological diagnosis and judged on whether gastric cancer invaded the serosa. The sensitivity, specificity, and accuracy of EUS and multiphoton imaging were compared. After the final H&E pathological diagnosis was revealed, the sensitivity, specificity, and accuracy of EUS (Table 4) and multiphoton imaging (Table 5) for T4 gastric cancer were 70% and 90% (*p* = 0.029), 66.67% and 96.67% (*p* = 0.003), and 68.33% and 93.33% (*p* = 0.001), respectively (Table 6). Multiphoton imaging significantly improved the diagnostic accuracy for T4 gastric cancer compared with EUS.

## Discussion

Last year, Spolverato G reported that the tumor stage on EUS did not often correlate with the T stage on final pathological analysis and that EUS should be combined with other staging modalities to optimize the staging of patients with gastric cancer^10^. Fairweather M also reported that EUS and CT was inadequate in identifying individual T stages and that a combined staging approach is required for accurate staging of patients with gastric cancer^5^. This year, Serrano OK reported that EUS appeared to correlate poorly with pathology in the preoperative staging of gastric cancer, which limited its utility in the neoadjuvant setting^11^. A previous study also found that the concordance between EUS and pathologic results was lower than expected for individual T stages^12^. In our study, the sensitivity, specificity, and accuracy of EUS for T4 gastric cancer were 70%, 66.67%, and 68.33%, respectively, which further confirmed that EUS was not sufficient to guide neoadjuvant therapy for patients with gastric cancer. Laparoscopy has become an effective staging tool for T4 gastric cancer, avoiding unnecessary laparotomy and improving the detection of peritoneal metastasis. However, there is no instrument that could achieve a real-time *in-situ* histopathological diagnosis of gastric cancer with serosal invasion. The limitations of the current medical procedures for real-time *in-situ* histopathological analysis of serosal invasion inspired the development of new diagnostic imaging modalities for the direct microscopic visualization of serosal invasion. In this study, we established the optical diagnostic features of gastric cancer with or without serosal invasion using multiphoton imaging and compared the sensitivity, specificity, and accuracy of EUS and multiphoton imaging for T4 gastric cancer. Our results showed that the sensitivity, specificity, and accuracy of EUS and multiphoton imaging for T4 gastric cancer were 70% and 90% (*p* = 0.029), 66.67% and 96.67% (*p* = 0.003), and 68.33% and 93.33% (*p* = 0.001), respectively. Multiphoton imaging significantly improved the diagnostic accuracy for T4 gastric cancer compared with EUS. Multiphoton imaging allows for subcellular resolution imaging of intrinsic fluorescence and SHG from unprocessed tissue with minimal optical attenuation and photo-damage. Multiphoton imaging clearly showed micro-anatomical differences between gastric cancer with and without serosal invasion similar to standard histopathological imaging but without the need for tissue processing, which supports the application of multiphoton laparoscopy for the real-time *in situ* imaging of gastric cancer with or without serosal invasion. Moreover, multiphoton imaging could quantify the collagen content and show the change of collagen when cancer cells invade the gastric serosa; this feature is a large improvement compared with the current, routine H&E images and collagen Masson images. In our study, the MPM system showed that the sample imaging could be recorded up to 150 μm deep, which is enough to distinguish normal serosa and abnormal serosa invaded by gastric cancer cells. From the serosal side, multiphoton imaging revealed collagen loss and degradation as well as cellular and nuclear pleomorphism in gastric cancer with serosal invasion. Our 2D image and 3D-stacking image show that MPM is advantageous for making a real-time optical diagnosis of gastric cancer with or without serosal invasion.

Recently, several groups have developed forms of miniature multiphoton endoscopes or sub-mm-diameter probes^13^,^14^,^15^,^16^, and another group has developed new photonic crystal fibers to allow delivery of 100-femtosecond pulses through optical fibers with more than enough power for multiphoton microscopy and multiphoton endoscopy^17^. More recently, clinical multiphoton tomography with two-photon microendoscopy, such as DermaInspect, has provided a powerful tool for the non-invasive *in vivo* examination of skin tissue^18^. Presently, multiphoton tomography is applied in clinical dermatology^19^. Jain M. *et al.* reported that multiphoton microscopy is a potential intraoperative tool for the detection of carcinoma *in situ* in human bladders^20^. Although multiphoton imaging could not currently be used as the real-time intrabody imaging technique, the ultimate value of this technique would be a non-destructive, *in vivo* evaluation of tissue, which means performing an optical diagnosis of tissue by visual examination with endoscopy or laparoscopy. Recent advances in multiphoton instrumentation and analysis tools might allow this technique to incorporated into the clinical setting for cancer diagnosis^21^. These advances would require collaboration with commercial instrumentation companies and also with surgeons and pathologists to adopt these new methods. The clinical use of multiphoton imaging techniques might greatly enhance the diagnosis and treatment of gastric cancer. We stress that multiphoton imaging would not replace classical histology but rather would provide additional metrics to pathologists and surgeons for bedside determination of cancer diagnosis and treatment.

In the clinic, EUS is applied from the mucosal side and is inadequate for identifying individual T stages. As we mentioned above, several studies reported that EUS correlated poorly with pathology in the preoperative staging of gastric cancer^5^,^10^,^11^,^12^. Therefore, laparoscopy is routinely used to determine whether gastric serosa has been invaded by cancer cells. For gastric cancer with serosal invasion, neoadjuvant therapy is required to downstage the tumor. Currently, laparoscopy could not achieve a real-time *in-situ* histopathological diagnosis for gastric cancer with serosal invasion, which is why we performed this study to compare MPM and EUS for T4 gastric cancer. Our results showed that multiphoton imaging significantly improved the diagnostic accuracy for T4 gastric cancer compared with EUS. Our final purpose is to integrate MPM into laparoscopy to perform real-time “optical biopsies” for gastric cancer from the serosal side. This study provides the groundwork for further use of multiphoton laparoscopy to perform a real-time non-invasive optical diagnosis for gastric cancer with or without serosal invasion. Because the aim is to improve the diagnostic accuracy for T4 gastric cancer in the clinic, regardless of the method and side, it is not necessary to compare both techniques from the serosal side. We have good reason to believe that it is feasible to use multiphoton imaging to evaluate neoadjuvant therapy for gastric cancer with serosal invasion. With miniaturization and integration of sub-mm-diameter probes and laparoscopy, multiphoton imaging has the potential to provide real-time “optical biopsies” for gastric cancer with or without serosal invasion in the near future.

## Patients and Methods

### Study design

There were two stages to this study. First, a pilot study was performed to establish the optical diagnostic features of gastric cancer with or without serosal invasion using multiphoton imaging. Twenty fresh, unfixed, and unstained full-thickness gastric specimens underwent multiphoton imaging followed by routine pathological procedures and Masson’s trichrome staining for collagen. The multiphoton images were compared with gold standard H&E stained images as well as Masson’s trichrome stained images. Second, a blinded study was performed to compare the sensitivity, specificity, and accuracy of multiphoton imaging and EUS for T4 gastric cancer by investigating 60 cases. The Institutional Review Board of Nanfang Hospital approved this study. All of the experiments were performed in accordance with the approved guidelines.

### Patients

Patients with gastric cancer, which was confirmed by a preoperative endoscopic biopsy, were recruited to participate in this study. Written informed consent was obtained prior to study participation. The inclusion criteria for this study included an age greater than 18 years, the ability to provide informed consent, an American Society of Anesthesiologists (ASA) class 1–3, and a gastric cancer suitable for radical resection. The exclusion criteria included the presence of bleeding, obstruction, or perforation, an ASA class 4–5, a gastric cancer with distant metastasis, or if the patient was currently receiving or had received neoadjuvant therapy. Eighty cases underwent gastrectomy in this study between June 2013 and December 2015.

### Specimens

In the pilot study, twenty fresh, unfixed, and unstained gastric specimens were investigated. After being removed by surgeons, each gastric specimen was kept in a standard pathologic transport container covered with ice and then sent to the multiphoton microscopy (MPM) lab. The serosal side of each sample was examined using multiphoton imaging, and then each sample went through routine pathological procedures, which included 10% buffered formalin processing, paraffin embedding, sectioning at 5 µm, and finally H&E staining. To evaluate tissue collagen, Masson’s trichrome staining was used. Multiphoton images, H&E staining images, and Masson’s trichrome staining images were analyzed and compared by the same attending pathologist, and then the multiphoton diagnostic criteria were established.

In the blinded study, 60 patients with gastric cancer, confirmed by endoscopic biopsy, were recruited to participate in this study. Each patient underwent preoperative EUS and then underwent laparoscopic radical gastrectomy with D2 lymphadenectomy. Each gastric specimen was kept in a standard pathologic transport container and then sent to the MPM lab. The serosal side of each sample was examined using multiphoton imaging, and then each sample went through routine pathological procedures. The multiphoton images were analyzed and diagnosed by the attending pathologist who established the multiphoton diagnostic features. The EUS doctor and the attending pathologist who analyzed the multiphoton images were blinded to the final H&E pathological diagnosis. The sensitivity, specificity, and accuracy of the multiphoton imaging and EUS were compared after the final H&E pathological diagnosis was revealed.

### Multiphoton imaging

After being cleaned with 0.9% saline solution, each gastric specimen was placed on the microscope slide, and the serosal side underwent MPM examination. The serosal layer was our region of interest. Because each imaging session was approximately 5 min in duration, it was not necessary to drip phosphate buffered saline solution onto the sample to avoid dehydration and shrinkage. Multiphoton imaging has the ability to achieve deep penetration, and high-resolution images could be obtained up to depths of several hundred microns. Therefore, multiphoton imaging could completely show serosal changes in gastric cancer. Multiphoton imaging takes approximately 5 min, whereas the routine pathological procedure usually takes 3 days. The MPM system used in this study has been described previously^22^. This system used a high-throughput scanning inverted Axiovert 200 microscope (Zeiss LSM 510 META, Jena, Germany) and a mode-locked femtosecond Titanium:sapphire (Ti:s) laser (110 fs, 76 MHz) tunable from 700 nm to 980 nm (Coherent Mira 900-F, Coherent, Inc., Santa Clara, CA, USA). For high-resolution imaging, a high numerical aperture oil immersion objective (Plan-Apochromat 63×, N.A.1.4; Zeiss) was employed in the MPM examination. The META detector collected the backward multiphoton signals from the tissue sample. Specifically, two channels were used: one channel (430–708 nm, green color-coded) was used to collect TPA signals, whereas the other channel (387–410 nm, red color-coded) was used to record SHG signals. The excitation wavelength (λ_*ex*_) used in this study was 800 nm. All the images had a 12-bit pixel depth. The images were obtained at 2.56 μs per pixel.

### Quantification of Collagen

To further quantify the changes in collagen in gastric cancer with or without serosal invasion, three SHG images from each specimen were selected for quantitative analysis. In this work, two analyses were performed. First, the collagen content was measured by counting the ratio of the SHG pixels to total pixels. Second, the grey-level co-occurrence matrix texture module of ImageJ software was used to analyze the collagen structure, as reported previously^23^,^24^. Briefly, texture features could be extracted by the use of grey-level statistical patterns between neighboring pixels. The correlation feature, a measure of intensity correlation as a function of pixel distance, relates to the collagen structure by indicating fiber and separation. Similar to the previous work^23^, a correlation value at the distance of 30 pixels is defined as the collagen structure, and a loss of collagen structure leads to a large correlation value.

### Sample size determination in the blinded study

Currently, the accuracy of EUS for gastric cancer T-staging is from 41% to 74.7%^5^,^11^,^12^,^25^,^26^. In the blinded study, the endpoint is T4-staging diagnostic accuracy between EUS and multiphoton imaging. In our institution, the EUS accuracy for T4 gastric cancer was 70% according to previous data. We hypothesized that the T4-staging diagnostic accuracy of multiphoton imaging is 90%, and therefore, 60 cases were included. With the number of cases that were used, the study would have an 80% power to detect a 20% increase in the accuracy of T4-staging by multiphoton imaging (two-sided type I error = 0.05).

## Additional Information

**How to cite this article**: Yan, J. *et al.* Real-time optical diagnosis of gastric cancer with serosal invasion using multiphoton imaging. *Sci. Rep.* **6**, 31004; doi: 10.1038/srep31004 (2016).

## Figures and Tables

**Table 1:** Patient demographics and cancer characteristics in the pilot study (20 cases).

Variable	Gastric cancer without serosal invasion (10 cases)	Gastric cancer with serosal invasion (10 cases)
Age (years): Median (range)	50 (39–68)	55 (41–67)
Gender (Male/Female)	8/2	7/3
Body mass index (kg/m^2^): Median (range)	23 (21–26)	22 (19–24)
ASA class (1/2/3)	0/3/7	0/4/6
Cancer size (cm): Median (range)	3 (1–5)	6 (4–8)
Cancer location (upper/middle/lower)	2/2/6	1/2/7
Tumor differentiation		
Well differentiated	1	0
Moderately differentiated	3	3
Poorly differentiated	6	7
Surgical procedure		
Subtotal gastrectomy	7	6
Total gastrectomy	3	4
AJCC/UICC stage (I/II/III/IV)	1/7/2/0	0/0/9/1

Abbreviations: AJCC, American Joint Committee on Cancer; UICC, Union for International Cancer Control.

**Table 2:** Patient demographics and cancer characteristics in the blinded study (60 cases).

Variable	Patients with gastric cancer (60 cases)
Age (years): Median (range)	54 (31–69)
Gender (Male/Female)	36/24
Body mass index (kg/m^2^): Median (range)	24 (21–27)
ASA class (1/2/3)	0/23/37
Cancer size (cm): Median (range)	4 (2–7)
Cancer location (upper/middle/lower)	11/14/35
EUS T-staging (T1/T2/T3/T4)	6/13/10/31
Tumor differentiation	
Well differentiated	2
Moderately differentiated	28
Poorly differentiated	30
Surgical procedure	
Subtotal gastrectomy	41
Total gastrectomy	19
AJCC/UICC stage (I/II/III/IV)	6/14/37/3

Abbreviations: AJCC, American Joint Committee on Cancer; UICC, Union for International Cancer Control.

**Table 3:** Optical diagnostic features of gastric cancer with or without serosal invasion using multiphoton imaging.

	Multiphoton imaging	
	Two-photon-excited fluorescence	Second harmonic generation
Non-serosal invasion	1. Regular elastic fibers	3. Regular collagen structure
	2. No cellular and nuclear pleomorphism	4. Correlation value of 0.21 ± 0.05
		5. Collagen content of 0.79 ± 0.16
Serosal invasion	1. Cellular and nuclear pleomorphism	4. Irregular collagen structure
	2. Irregular tubular structures	5. Correlation value of 0.69 ± 0.18
	3. Cancer cells characterized by irregular size and shape, enlarged nuclei, and increased nuclear-cytoplasmic ratio	6. Collagen significantly decreased, collagen content of 0.36 ± 0.18

**Table 4:** The accuracy of EUS for T4 gastric cancer.

		Pathology		
N = 60		Serosal invasion (N1 = 30)	Non- serosal invasion (N2 = 30)	
EUS	T4 (N3 = 31)	21	10	PPV = 67.74% (21/31)
	Non- T4 (N4 = 29)	9	20	NPV = 68.97% (20/29)
		Sens = 70% (21/30)	Spec = 66.67% (20/30)	Accuracy = 68.33% (41/60)

Abbreviations: EUS, endoscopic ultrasonography; Sens, sensitivity; Spec, specificity; PPV, positive predictive value; NPV, negative predictive value.

**Table 5:** The accuracy of multiphoton imaging for T4 gastric cancer.

		Pathology		
N = 60		Serosal invasion (N1 = 30)	Non- serosal invasion (N2 = 30)	
Multiphoton imaging	T4 (N3 = 28)	27	1	PPV = 96.43% (27/28)
	Non- T4 (N4 = 32)	3	29	NPV = 90.63% (29/32)
		Sens = 90% (27/30)	Spec = 96.67% (29/30)	Accuracy = 93.33% (56/60)

Abbreviations: Sens, sensitivity; Spec, specificity; PPV, positive predictive value; NPV, negative predictive value.

**Table 6:** Comparison between endoscopic ultrasonography and multiphoton imaging.

	Endoscopic ultrasonography	Multiphoton imaging	*P* value
Sensitivity	70% (21/30)	90% (27/30)	0.029
Specificity	66.67% (20/30)	96.67% (29/30)	0.003
Accuracy	68.33% (41/60)	93.33% (56/60)	0.001
Positive predictive value	67.74% (21/31)	96.43% (27/28)	0.005
Negative predictive value	68.97% (20/29)	90.63% (29/32)	0.035
